# Differential Proteomic Analysis Using iTRAQ Reveals Alterations in Hull Development in Rice (*Oryza sativa* L.)

**DOI:** 10.1371/journal.pone.0133696

**Published:** 2015-07-31

**Authors:** Shuzhen Wang, Wenyue Chen, Wenfei Xiao, Changdeng Yang, Ya Xin, Jieren Qiu, Weimin Hu, Wu Ying, Yaping Fu, Jianxin Tong, Guocheng Hu, Zhongzhong Chen, Xianping Fang, Hong Yu, Wenguo Lai, Songlin Ruan, Huasheng Ma

**Affiliations:** 1 Laboratory of Plant Molecular Biology & Proteomics, Institute of Biotechnology, Hangzhou Academy of Agricultural Sciences, Hangzhou 310024, China; 2 State Key Laboratory of Rice Biology, China National Rice Research Institute, Hangzhou 310006, China; 3 Department of Agronomy, College of Agriculture and Biotechnology, Zhejiang University, Hangzhou 310012, China; CSIR-Institute of Genomics and Integrative Biology, INDIA

## Abstract

Rice hull, the outer cover of the rice grain, determines grain shape and size. Changes in the rice hull proteome in different growth stages may reflect the underlying mechanisms involved in grain development. To better understand these changes, isobaric tags for relative and absolute quantitative (iTRAQ) MS/MS was used to detect statistically significant changes in the rice hull proteome in the booting, flowering, and milk-ripe growth stages. Differentially expressed proteins were analyzed to predict their potential functions during development. Gene ontology (GO) terms and pathways were used to evaluate the biological mechanisms involved in rice hull at the three growth stages. In total, 5,268 proteins were detected and characterized, of which 563 were differentially expressed across the development stages. The results showed that the flowering and milk-ripe stage proteomes were more similar to each other (*r*=0.61) than either was to the booting stage proteome. A GO enrichment analysis of the differentially expressed proteins was used to predict their roles during rice hull development. The potential functions of 25 significantly differentially expressed proteins were used to evaluate their possible roles at various growth stages. Among these proteins, an unannotated protein (Q7X8A1) was found to be overexpressed especially in the flowering stage, while a putative uncharacterized protein (B8BF94) and an aldehyde dehydrogenase (Q9FPK6) were overexpressed only in the milk-ripe stage. Pathways regulated by differentially expressed proteins were also analyzed. Magnesium-protoporphyrin IX monomethyl ester [oxidative] cyclase (Q9SDJ2), and two magnesium-chelatase subunits, ChlD (Q6ATS0), and ChlI (Q53RM0), were associated with chlorophyll biosynthesis at different developmental stages. The expression of Q9SDJ2 in the flowering and milk-ripe stages was validated by qRT-PCR. The 25 candidate proteins may be pivotal markers for controlling rice hull development at various growth stages and chlorophyll biosynthesis pathway related proteins, especially magnesium-protoporphyrin IX monomethyl ester [oxidative] cyclase (Q9SDJ2), may provide new insights into the molecular mechanisms of rice hull development and chlorophyll associated regulation.

## Introduction

Rice (*Oryza sativa* L.) supports nearly half the world population and is one of the world’s most important grain crops [1–4]. The mature rice seed consists of caryopsis and hull. The rice caryopsis contains the caryopsis coat, aleurone, embryo and starchy endosperm. The aleurone layer is the outermost layer of the endosperm, followed by the inner starchy endosperm [5]. The embryo consists of the scutellum, embryonic axis and various sheathing structures. The starchy endosperm, accounting for over 80% of the caryopsis, contains parenchyma cells filled with reserves [6]. The rice hull (husk), the outer cover of the rice grain, is an important floral organ comprised of lemma and palea. The lemma covers two-thirds of the seed, while the palea is the lining that hugs the seed. The hull is indigestible because of the presence of opaline silica (a hydrated form of silica) and lignin, a complex component that helps to hydrate the rice grain. Rice hull not only acts as a mechanical barrier to prevent damage and maintain humidity for the developing seed, but is also a critical factor that affects yield and milling quality of the grain in terms of its volume, shape, and size [7–12].

Genetic studies and gene cloning have helped understand the functions of the large numbers of genes involved in rice hull development. For example, a rice mutant with twisted hull (*twh*) derived from a breeding population of rice was reported to exhibit reduced grain weight compared with the wild type parent [13]. Similarly, a rice floral organ mutant (*bh1*) has a beak-shaped hull, which was found to have a negative effect on grain yield [14]. The stunted lemma palea 1 rice mutant (*slp1*) displays severely degenerated lemmas/paleae, and *SLP1* was reported to be localized in a 46.4-kb genomic region that contained three putative genes, *OsSPL16*, *OsMADS45*, and *OsMADS37* [15]. A palea formation controlling gene, depressed palea 1 (*dp1*), encodes a nuclear-localized AT-hook DNA-binding protein that causes a primary defect in the main structure of the palea, which is required for palea formation and floral organ number control [16]. The rice MADS-box factor (*OsMADS1*) is an early-acting regulator of inner floral organs that controls the differentiation of specific cell types in the lemma and palea [17]. Li and coworkers found that *TH1* accumulated mainly in young inflorescence and was important in controlling lemma and palea development in rice [8]. Other genes that have been reported to be associated with rice hull development include frizzy panicle (*FZP*) [18], abnormal hull (*ah*) [19], sterile lemma (*G1*) [20], and elongated empty glume (*ELE*) [21]. Nevertheless, the genetic regulation of rice hull development has not yet been established. Systematic genome-wide analysis may provide further insights into the molecular genetic mechanisms associated with rice hull development.

Microarray technologies are useful for high-throughput gene expression analysis [22]. However, mRNA expression levels may not translate precisely into protein function and, therefore, mRNA profiling may not fully characterize the functional proteome. Proteomic research areas are currently mainly focused on abiotic stress and biotic stress in rice [23], the lack of a validated, high-throughput, quantitative functional proteomics platform for rice hull remains a major barrier to the identification of the underlying molecular mechanisms associated with its development.

The isobaric tags for relative and absolute quantification (iTRAQ) method has been developed for the simultaneous quantitative comparison and analysis of protein expression profiles of multiple samples [24–26]. Quantitative proteomic mass spectrometry methodologies can be classified into three categories: label-free (non-labeled), gel-based, and label-based. The labeled iTRAQ technique, which uses isotopic label-based protocols, yields very small coefficients of variation in quantitative measurements and is considered one of the most robust techniques for differential quantitative proteomic analyses [27, 28]. In the present study, we applied label-based iTRAQ to analyze quantitatively the alternations of the hull proteome in the booting stage, flowering stage, and milk-ripe stage of hull development. The aim was to identify differentially expressed proteins of the rice hull at various growth stages. The results provide a global insight into changes of the rice hull proteome during development.

## Materials and Methods

### Sample collection

A tested super-hybrid rice variety (*O*. *sativa* cv. LYP9) was supplied by the Wu Wang Nong Seed Group (Hangzhou, Zhejiang Province, China). Four replicates of 100 seeds were soaked in water for 24 h at 30°C to accelerate hydration and then transferred to germination boxes (18 cm × 13 cm × 10 cm) containing two layers of blotters moistened with distilled water. The seeds were germinated in a chamber for 1 d at 30°C to accelerate germination. Germinating seeds were sown in paddy soil on 14 June 2011. The rice plants started heading on 10 September 2011. Fresh hull samples at three different developmental stages, booting stage (PK1), flowering stage (PK2), and milk-ripe stage (PK3), were collected during heading and stored at -80°C until protein preparation.

### Protein preparation

One gram fresh weight of hulls was ground in liquid nitrogen and suspended in 5 ml of 10% (w/v) trichloroacetic acid in acetone with 0.07% (w/v) β-mercaptoethanol at -20°C for 1 h, followed by centrifugation for 15 min at 35,000 × *g*. The pellets were resuspended in acetone with 0.07% (w/v) β-mercaptoethanol and incubated at -20°C for 1 h, and then centrifuged for 15 min at 4°C. This step was repeated three times, after which the pellets were lyophilized. The crude protein power was solubilized in lysis buffer (8 M urea, 2 M thiourea, 4% CHAPS, 0.5% ampholine (pH 3–10), 50 mM DTT, and 1 mM PMSF) for 1 h at room temperature, followed by centrifugation for 15 min at 15,000 × *g*. The supernatant was collected in a 1.5-ml tube, and a 40-μl sample was taken to determine the protein concentration using the Bradford assay with bovine serum albumin as the standard.

### Protein digestion and iTRAQ labeling

Protein digestion was performed according to the FASP procedure [29] and the resulting peptide mixture was labeled using the 4-plex iTRAQ reagent according to the manufacturer’s instructions (Applied Biosystems, Boston, MA, USA). Briefly, 200 μg of protein for each sample was incorporated into 30 μl STD buffer (4% SDS, 100 mM DTT, 150 mM Tris-HCl pH 8.0). The detergent DTT and other low-molecular-weight components were removed using UA buffer (8 M Urea, 150 mM Tris-HCl pH 8.0) by repeat ultrafiltration (Microcon units, 30 kD). Then 100 μl 0.05 M iodoacetamide in UA buffer was added to block reduced cysteine residues and the samples were incubated for 20 min in the dark. The filters were washed three times with 100 μl UA buffer, and then twice with 100 μl DS buffer (50 mM triethylammoniumbicarbonate at pH 8.5). Finally, the protein suspensions were digested with 2 μg trypsin (Promega, Madison, WI, USA) in 40 μl DS buffer overnight at 37°C, and the resulting peptides were collected as a filtrate. The peptide content was estimated by UV light spectral density at 280 nm using an extinctions coefficient of 1.1 of 0.1% (g/l) solution that was calculated based on the frequency of tryptophan and tyrosine in vertebrate proteins.

For labeling, each iTRAQ reagent was dissolved in 70 μl of ethanol and added to the respective peptide mixture. The PK1 sample was divided into two parts that were labeled as (PK1)-114 and (PK1)-115. The sample that was labeled as (PK1)-114 was served as the control. The remaining samples were labeled as (PK2)-116 and (PK3)-117. All labeled samples were multiplexed and vacuum dried.

### Peptide fractionation with strong cation exchange (SCX) chromatography

The iTRAQ labeled peptides were fractionated by SCX chromatography using an AKTA Purifier system (GE Healthcare, London, UK). The dried peptide mixture was reconstituted and acidified with 2 ml buffer A (10 mM KH_2_PO_4_ in 25% of acetonitrile, pH 2.7) and loaded onto a PolySulfoethyl 4.6 x 100 mm column (5 μm, 200 Å, PolyLC Inc, Colombia, MD, USA). The peptides were eluted at a flow rate of 1 ml/min with a buffer B (500 mM KCl, 10 mM KH_2_PO_4_ in 25% acetonitrile, pH 2.7) gradient of 0%–10% for 2 min, 10%–20% for 25 min, 20%–45% for 5 min, and 50%–100% for 5 min. The elution was monitored by absorbance at 214 nm, and fractions were collected every 1 min. The collected fractions (about 30 fractions) were finally combined into 10 pools and desalted on C18 Cartridges (Empore SPE Cartridges C18 (standard density), bed I.D. 7 mm, volume 3 ml, Sigma, Santa Clara, CA, USA). Each fraction was concentrated by vacuum centrifugation and reconstituted in 40 μl of 0.1% (v/v) trifluoroacetic acid. All samples were stored at -80°C until analysis by liquid chromatography with tandem mass spectrometry (LC-MS/MS).

### LC-MS/MS analysis

From each fraction, 10 μg was trapped on a precolumn (200 μm x 0.5 mm) and then eluted on an analytical column (75 μm x 15 cm) for separation. Both columns were packed with Reprosil-Pur (RP) C18-AQ 3 μm 120Å phase (Eksigent, Dublin, CA, USA). The RP mobile phase A was 98% water (with 2% acetonitrile and 0.1% formic acid) while RP mobile phase B was 98% acetonitrile (with 2% H_2_O and 0.1% formic acid). The peptides were separated over 90 min using a linear gradient of 12%–30% of RP mobile B at a flow rate of 300 nl/min. The MS analysis was performed using a 5600 TripleTOF analyzer (QqTOF; AB SCIEX, Boston, MA, USA) in Information Dependent Mode. Precursor ions were selected across the mass range of 350–1250 m/z using 250 ms accumulation time per spectrum. A maximum of 30 precursors per cycle from each MS spectrum were selected for MS/MS analyses with 100 ms minimum accumulation time for each precursor and dynamic exclusion for 25 s. The MS/MS spectra were recorded in high sensitivity mode with rolling collision energy on and iTRAQ reagent collision energy adjustment on.

### Protein identification and relative quantitation criteria

Protein identification and relative iTRAQ quantification were performed with the Paragon algorithm [30] in the ProteinPilot Software 4.2 (AB SCIEX, Boston, MA), and the results were further processed using the Pro Group algorithm, where isoform-specific quantification was adopted to trace the differences between the expressions of various isoforms. Quantitative ratio of reporter ions, calculated by comparing the peak areas of each of these reporter ions in the mass spectrum, was used to evaluate the expression change of the protein in different samples. Normalization has been done during the quantification to correct experimental bias. Strict criteria were imposed to reduce the numbers of false positive expression changes. To accept proteins as showing differential expression between the different stages, we used the following criteria: 1) Proteins must have been identified in all four iTRAQ preparations; 2) Proteins must have been identified with greater than 95% confidence; 3) Proteins with 20% increase or decrease in replicated experiments (iTRAQ ratios (PK1)-115:(PK1)-114 <0.8 or >1.2) were excluded; and 4) Proteins with a ratio fold change >2 or <0.5 in expression (iTRAQ ratios (PK2)-116:(PK1)-114 or (PK3)-117:(PK1)-114 of >2 or <0.5) and a significant p-value (p <0.05) were considered to be differentially expressed.

### Protein mass spectrum hierarchical cluster analysis and statistical analysis

We selected 563 proteins from the 5,268 identified proteins for further analysis and the log_2_ ratios were normalized globally for each sample. To cluster the rice husk proteins of the three growth stages, we used Ward’s method, a hierarchical technique. Function heatmap.2 from the R package gplots was used to produce the graphical display of the dendrogram [31]. Pearson’s correlation coefficient and scatter plots were used to estimate the relationship between two random samples at various growth stages. Lowess was used to smooth the scatter plots by locally weighted regression. All the statistical analyses were performed in the R environment, using several CRAN packages (http://cran.r-project.org/).

### Gene ontology and pathway analysis

Gene ontology (GO) [32], UniProt [33], and GOEAST [34] were used to annotate the proteins under the biological process, molecular function, and cellular component GO categories in the booting, flowering, and milk-ripe stages of rice hull development. Rice gene annotations were taken from the Rice Annotation Project Database (RAP-DB) [35] and the Michigan State University (MSU) Rice Genome Annotation [36] website. Circos diagrams [37] were also used to analysis the differences at various growth stages. Proteins that were differentially expressed in the booting stage were analyzed using UniPathway [38] to identify molecular pathways that may be different in this growth stage compared with the other two stages.

### Expression validation by quantitative RT-PCR

Quantitative RT-PCR (qRT-PCR) assays were performed to validate the different expression levels of differentially expressed chlorophyll biosynthesis related proteins in the booting, flowering and milk-ripe growth stages. Relative gene expression levels were quantified based on the cycle threshold (Ct) values and normalized to the reference gene Actin (GenBank: AY212324). Each sample was repeated three times and the gene expression levels were calculated by the 2-^ΔΔCt^ method.

## Results

### Phenotype of rice hull at various growth stages

**Fig 1:**
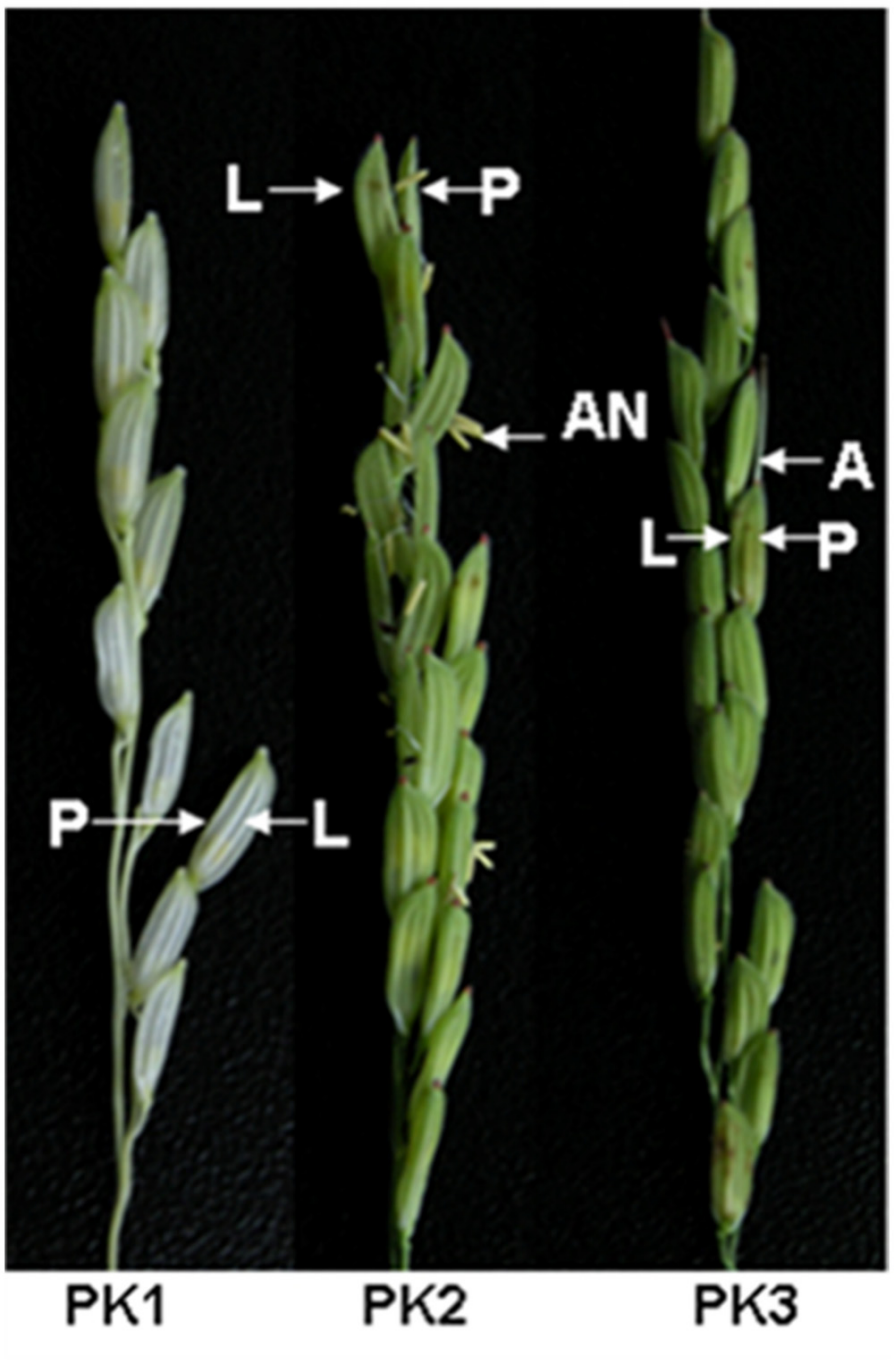
Phenotype of rice hull in three growth stages. A, awn; AN, anther; L, lemma; P, palea; PK1, booting stage; PK2, flowering stage; PK3, milk-ripe stage.

Rice hull samples were collected at three stages of husk development (Fig 1). The samples collected at the earliest stage, the booting stage, were characterized by swelling of the leaf sheath and elongated internodes. The samples collected at the flowering stage were selected between the opening and closing of the rice flower. The milk-ripe stage samples exhibited starch grains developing in the kernels that were soft and in the interior of the kernel.

### Protein identification and relative expression

Overall, we identified 5268 distinct proteins that had an estimated global False Discovery Rate (FDR) of <0.01. Only proteins that were identified with ≥95% confidence were considered. Among the 5,268 proteins, 563 differentially expressed proteins were identified for further analysis (S1 Table). Compared with their expressions in the booting stage, 235 proteins were upregulated and 326 were downregulated in the flowering stage, and 221 proteins were upregulated and 342 were downregulated in the milk-ripe stage.

### Quantitative comparison of protein expression in the three developmental stages

**Fig 2:**
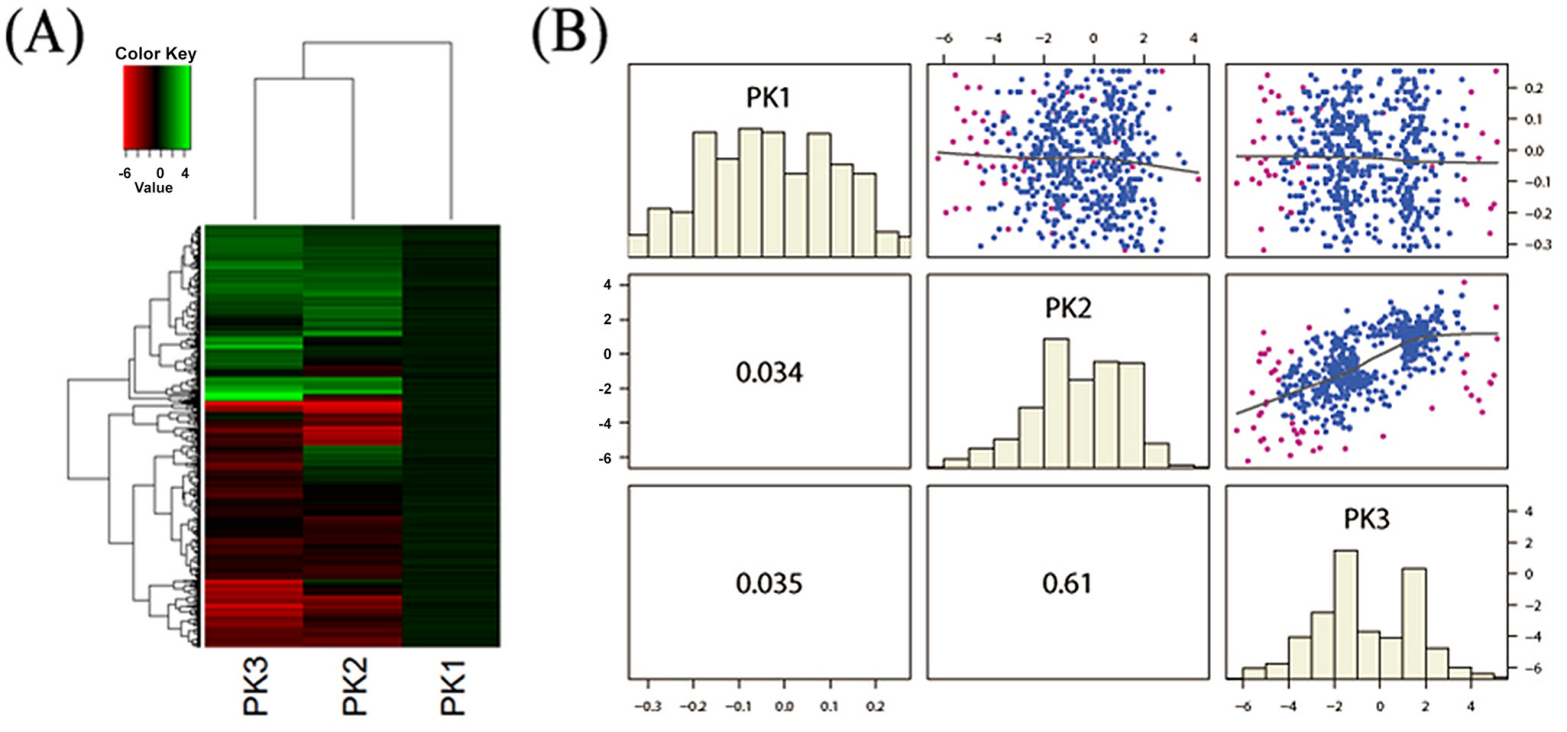
Quantitative comparison of protein expression in the three developmental stages. (A) Hierarchical clustering analysis of protein expression in three growth stages of rice hull. (B) Protein expression correlations in three growth stages of rice hull. PK1, booting stage; PK2, flowering stage; PK3, milk-ripe stage.

To investigate the expression levels in the rice hull proteomes at various growth stages, we used Ward’s method to perform unsupervised hierarchical clustering analysis of the 563 differentially expressed proteins. The samples were clustered based on the overall similarity in their protein expression patterns and the relationships were summarized in a dendrogram (Fig 2A). Results showed that the protein expression patterns in the flowering and milk-ripe stage proteomes were more similar to each other than either was to the protein expression patterns in the booting stage proteome. In both the flowering and milk-ripe stages the protein expression patterns had very high correlation coefficients (*r* = 0.61), while the correlation coefficient between the flowering or milk-ripe stages and the booting stage were lower (*r* = 0.034 and *r* = 0.035, respectively) (Fig 2B).

### Overall gene ontology analysis

**Fig 3:**
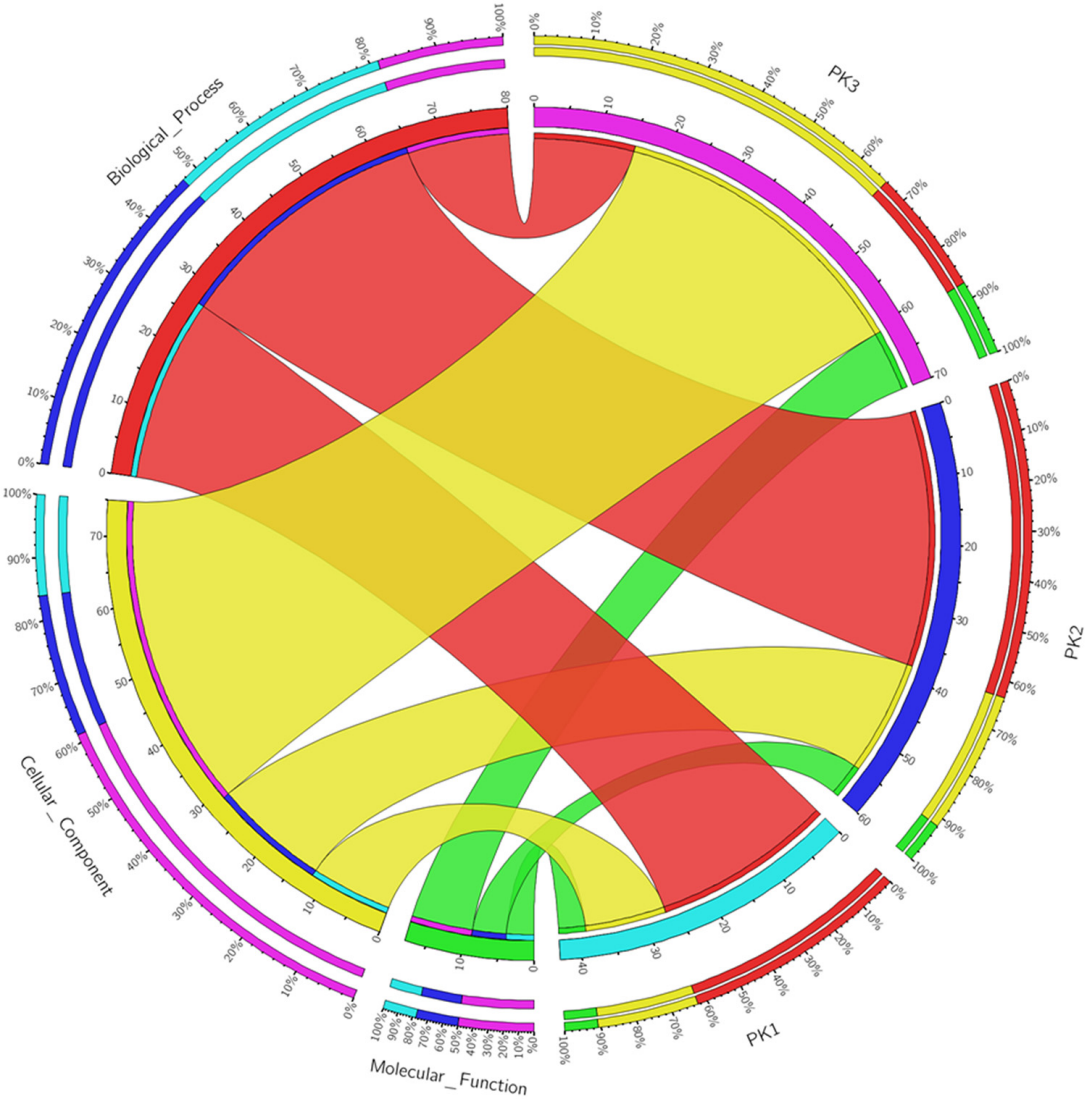
Gene ontology analysis of differentially expressed proteins in three growth stages of rice hull. PK1, booting stage; PK2, flowering stage; PK3, milk-ripe stage.

To obtain a global picture of the proteomic changes during rice hull development, the differentially expressed proteins were annotated with GO terms and a GO functional analysis was performed. We found that that 27 biological process terms, four molecular function terms, and 12 cellular component terms were statistically enriched in the booting stage. In the flowering stage, 38 biological process terms, five molecular function terms, and 17 cellular component terms were statistically enriched; while in the milk-ripe stage, 15 biological process terms, nine molecular function terms, and 46 cellular component terms were statistically enriched (Fig 3, S2 Table). The numbers of GO terms in the three categories were more similar in the booting and flowering stages than either was in the milk-ripe stage. Compared with cellular component terms (S1 Fig) and biological process terms (S2 Fig), fewer significant molecular function terms were involved and are more likely to characterize the differentially expressed proteins in the booting, flowering and milk-ripe growth stages.

### Molecular function analysis

**Fig 4:**
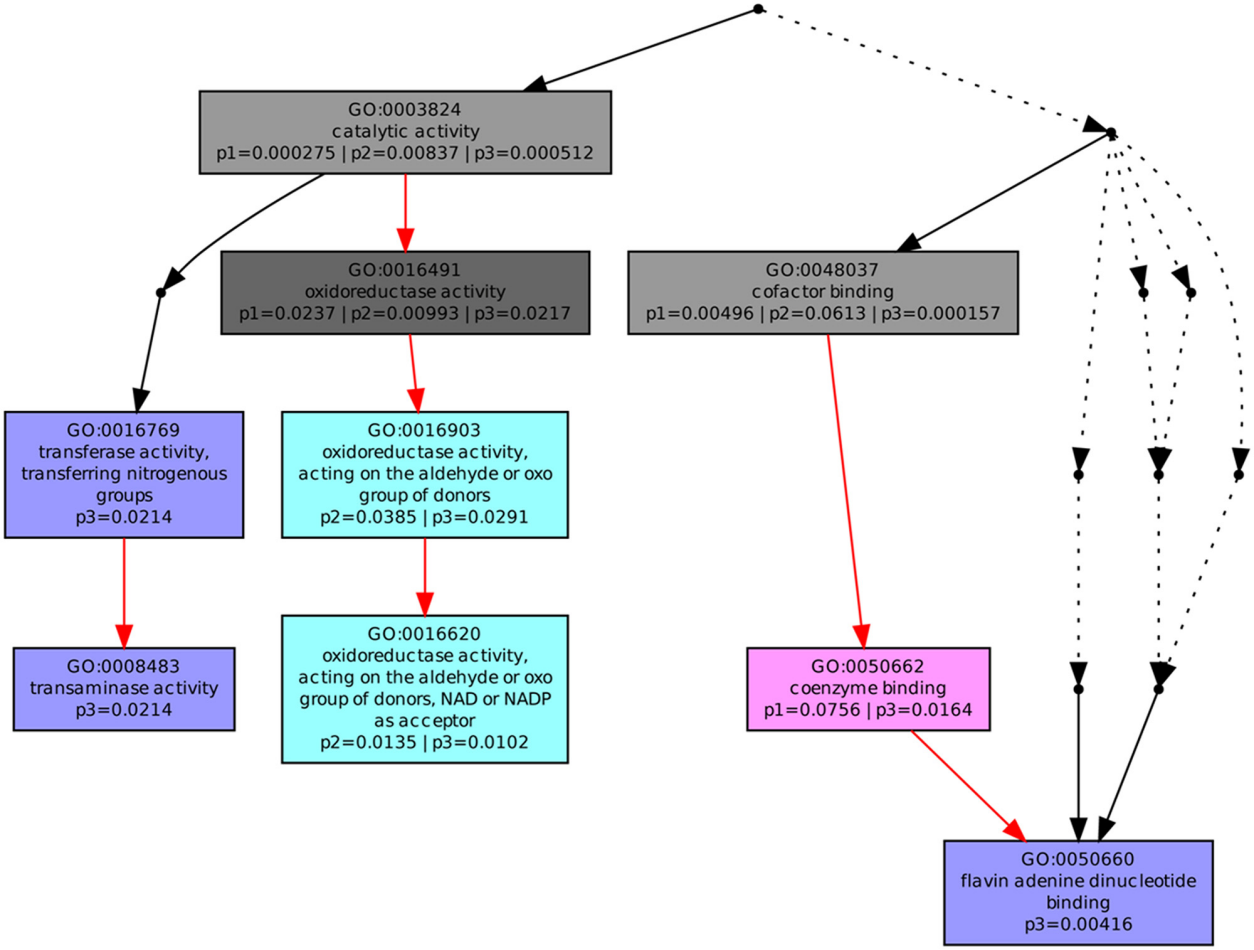
Molecular function analysis of differentially expressed proteins in three growth stages of rice hull. P1, booting stage; P2, flowering stage; P3, milk-ripe stage.

Nine highly enriched molecular function GO terms were associated with all three development stages (Fig 4). Catalytic activity, oxidoreductase activity, and cofactor binding were common to rice hull development in the three growth stages, although the numbers of proteins annotated with these terms decreased in the flowering stage and increased in the milk-ripe stage. Proteins annotated as coenzyme binding (GO:0050662) were present in the booting stage (P = 0.0756) and milk-ripe stage (P = 0.0164), while proteins annotated as oxidoreductase activity, acting on the aldehyde or oxo group of donors, NAD or NADP as acceptor (GO:0016620), and oxidoreductase activity, acting on the aldehyde or oxo group of donors (GO:0016903) were found in the flowering and milk-ripe stages. The molecular function terms were also compared in the three development stages (Table 1). Overall 333, 93, and 126 proteins involved in catalytic activity were identified in the booting, flowering, and milk-ripe stages, respectively. The numbers of proteins related to oxidoreductase activity and cofactor binding followed the same trend at various growth stages; i.e., highest in the booting stage and lower in the other two stages. Twenty-five proteins annotated with five GO terms (GO:0016620, GO:0016903, GO:0008483, GO:0016769, and GO:0050660) were selected to evaluate the heterogeneity of the rice hull proteome at different growth stages. Among the proteins annotated with the two oxidoreductase activity related terms (GO:0016620 and GO:0016903), five proteins (glyceraldehyde-3-phosphate dehydrogenase 3, cytosolic (GAPC3; Q6K5G8); glyceraldehyde-3-phosphate dehydrogenase (Q8S4Y9); putative cytosolic aldehyde dehydrogenase (Q69XE0); aldehyde dehydrogenase ALDH2b (Aldh2b; Q9FRX7); and putative uncharacterized protein (B8AIJ7)) were common to the flowering and milk-ripe stages; one protein (Q7X8A1) was unique to the flowering stage, and two proteins (B8BF94 and Aldehyde dehydrogenase (ALDH; Q9FPK6)) were found only in the milk-ripe stage. Seventeen proteins unique to the milk-ripe stage were related to transaminase activity (GO:0008483), transferase activity, transferring nitrogenous groups (GO:0016769), and flavin adenine dinucleotide binding (GO:0050660). Seven of the proteins (alanine aminotransferase (Q9S768); aspartate aminotransferase (Q0JJ47); putative acetylornithine aminotransferase (Q688Q8); Q94EG1; aspartate aminotransferase (Q6KAJ2); B8ANL7, and Q5N9Z8) were annotated with two of the terms (GO:0008483 and GO:0016769), while the other ten proteins (A2WTU1; B8AYF2; B7E6V7; Q0DWI9; thioredoxin reductase (B8B5I4); Q0D8R4; putative ferredoxin-NADP(H) oxidoreductase (Q6ZFJ3); probable D-2-hydroxyglutarate dehydrogenase, mitochondrial (D2HGDH; Q7XI14); acetolactate synthase (ALS; A5X300); and putative CPRD2 (Q5Z952) were annotated with GO:0050660.

**Table 1:** Molecular function of related proteins in three growth stages of rice hull.

		Protein Number				
Molecular Function (GO ID)	Term		Unique/Total			P-value
		Shared^a^	PK1^b^	PK2^b^	PK3^b^	
GO:0003824	catalytic activity	57	171/333	0/93	0/126	0.003053581
GO:0016491	oxidoreductase activity	22	43/94	0/33	0/40	0.018451297
GO:0048037	cofactor binding	12	18/50	0/17	0/27	0.022130506
GO:0050662	coenzyme binding	18	17/35	-	0/18	0.046026777
GO:0016620	oxidoreductase activity, acting on the aldehyde or oxo group of donors, NAD or NADP as acceptor	5	-	1/6	2/7	0.011866133
GO:0016903	oxidoreductase activity, acting on the aldehyde or oxo group of donors	5	-	1/6	2/7	0.03380623
GO:0008483	transaminase activity	0	-	-	7/7	0.021352469
GO:0016769	transferase activity, transferring nitrogenous groups	0	-	-	7/7	0.021352469
GO:0050660	flavin adenine dinucleotide binding	0	-	-	10/10	0.00416116

^a^Shared indicates proteins with a shared molecular function in the existed significant molecular function of the three growth stages.

^b^PK1, booting stage; PK2, flowering stage; PK3, milk-ripe stage.

### Pathway analysis and qRT-PCR validation

**Fig 5:**
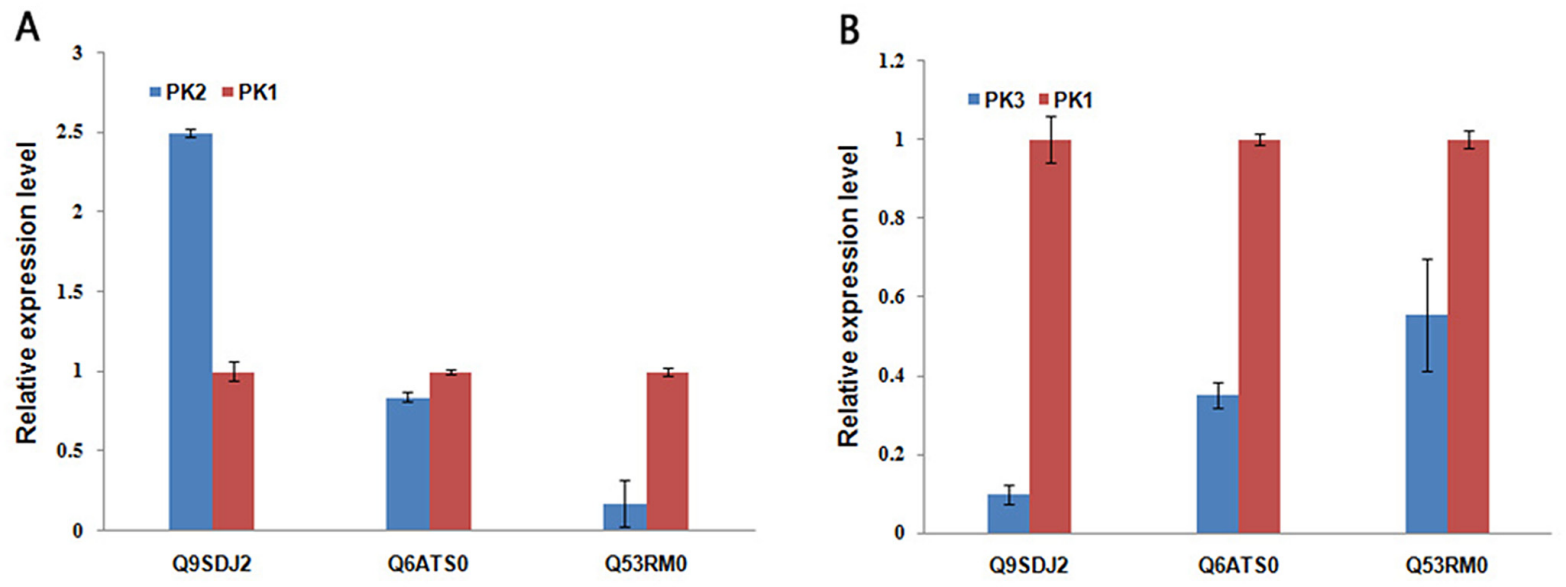
Validation of differentially expressed proteins in chlorophyll biosynthesis pathways. P1, booting stage; P2, flowering stage; P3, milk-ripe stage.

The iTRAQ results were also evaluated using UniPathway. Twenty-six proteins in the booting stage samples were assigned to 19 pathways, including sucrose biosynthesis, glycolysis, chlorophyll biosynthesis, and oxylipin biosynthesis. The regulation of proteins corresponding to the related pathways varied in the flowering and milk-ripe stages. Compared with the booting stage, six proteins were up-regulated and six were down-regulated in the flowering stage, while 11 proteins were up-regulated (Table 2) and 10 were down-regulated in the milk-ripe stage (Table 3). The expressions of GAPC3 (Q6K5G8) in the glycolysis pathway, phenylalanine ammonia-lyase (B7EFQ4) in the trans-cinnamate biosynthesis pathway, and the probable sucrose-phosphate synthase (Q0JGK4) in the sucrose biosynthesis pathway in the flowering and milk-ripe stages were obviously higher than in the booting stage. Compared with the booting stage, L-lactate dehydrogenase (Q0E4Q5) in the pyruvate fermentation to lactate pathway, S-adenosylmethionine synthase 2 (SAM2; P93438) in the S-adenosyl-L-methionine biosynthesis pathway, adenosylhomocysteinase (Q84VE1) in the L-homocysteine biosynthesis pathway, and chalcone synthase 1 (CHS1; Q2R3A1) in flavonoid biosynthesis pathway were all down-regulated in the flowering and milk-ripe stages. Three significantly differentially expressed proteins were found to be associated with chlorophyll biosynthesis pathways. The expression of magnesium-protoporphyrin IX monomethyl ester [oxidative] cyclase (Q9SDJ2) was significantly up-regulated in the flowering stage, and down-regulated in the milk-ripe stage. The two magnesium-chelatase subunits, ChlD (Q6ATS0) and ChlI (Q53RM0), were down-regulated in the milk-ripe stage (Table 3). Among these chlorophyll biosynthesis pathway related proteins, the expression of magnesium-protoporphyrin IX monomethyl ester [oxidative] cyclase (Q9SDJ2) in the flowering and milk-ripe stages was validated by qRT-PCR, while ChlD (Q6ATS0) and ChlI (Q53RM0) was validated down-regulated in the milk-ripe stage (Fig 5).

**Table 2:** Pathway analysis of differentially upregulated expressed proteins in the flowering and milk-ripe stages compared with the booting stage.

Pathway	Control (PK1)^a^	Upregulated (PK2)^a^	Upregulated (PK3)^a^
L-methionine biosynthesis via salvage pathway	F4MG97	F4MG97	
trans-cinnamate biosynthesis	Q0DZE3;B7EFQ4;Q6K6Q1	B7EFQ4	B7EFQ4;Q6K6Q1
chlorophyll biosynthesis (light-independent)	Q9SDJ2	Q9SDJ2	
chlorophyll biosynthesis	Q6ATS0;Q53RM0		
Pyruvate fermentation to lactate	Q0E4Q5		
Oxylipin biosynthesis	A2XL22		A2XL22
sucrose biosynthesis	Q0JGK4	Q0JGK4	Q0JGK4
S-adenosyl-L-methionine biosynthesis	P93438		
L-homocysteine biosynthesis	Q84VE1		
urea degradation	E0ZS48		E0ZS48
protoporphyrin-IX biosynthesis	Q10LR9		
tetrahydrofolate interconversion	Q7Y1F0;Q10BJ7	Q7Y1F0	Q10BJ7
flavonoid biosynthesis	Q2R3A1		
glutathione biosynthesis	Q6Z3A3		Q6Z3A3
pentose phosphate pathway	Q10M94		Q10M94
Glycolysis	Q6K5G8;Q7XKB5;Q7FAH2;B8ACJ0	Q6K5G8	Q6K5G8
AMP biosynthesis via de novo pathway	A2XD35		A2XD35
isopentenyl diphosphate biosynthesis via DXP pathway	Q5N8G1		Q5N8G1
chorismate biosynthesis	B8AKA5		B8AKA5

^a^PK1, booting stage; PK2, flowering stage; PK3, milk-ripe stage.

**Table 3:** Pathway analysis of differentially downregulated expressed proteins in the flowering and milk-ripe stages compared with the booting stage.

Pathway	Control (PK1)^a^	Downregulated (PK2)^a^	Downregulated (PK3)^a^
L-methionine biosynthesis via salvage pathway	F4MG97		F4MG97
trans-cinnamate biosynthesis	Q0DZE3;B7EFQ4;Q6K6Q1	Q0DZE3	
chlorophyll biosynthesis (light-independent)	Q9SDJ2		Q9SDJ2
chlorophyll biosynthesis	Q6ATS0;Q53RM0		Q6ATS0;Q53RM0
Pyruvate fermentation to lactate	Q0E4Q5	Q0E4Q5	Q0E4Q5
Oxylipin biosynthesis	A2XL22	A2XL22	
sucrose biosynthesis	Q0JGK4		
S-adenosyl-L-methionine biosynthesis	P93438	P93438	P93438
L-homocysteine biosynthesis	Q84VE1	Q84VE1	Q84VE1
urea degradation	E0ZS48		
protoporphyrin-IX biosynthesis	Q10LR9		Q10LR9
tetrahydrofolate interconversion	Q7Y1F0;Q10BJ7		
flavonoid biosynthesis	Q2R3A1	Q2R3A1	Q2R3A1
glutathione biosynthesis	Q6Z3A3		
pentose phosphate pathway	Q10M94		
Glycolysis	Q6K5G8;Q7XKB5;Q7FAH2;B8ACJ0		Q7XKB5;Q7FAH2;B8ACJ0
AMP biosynthesis via de novo pathway	A2XD35		
isopentenyl diphosphate biosynthesis via DXP pathway	Q5N8G1		
chorismate biosynthesis	B8AKA5		

^a^PK1, booting stage; PK2, flowering stage; PK3, milk-ripe stage.

## Discussion

Knowledge of proteomic changes that occur during rice hull development is important for understanding the mechanism that determines rice yield and quality. Many genetic and genomic resources have been used to elucidate details of the molecular mechanisms of rice hull development; however, until now, this information has been limited to a small number of genes [8, 13, 15–17] and was based mainly on genomic techniques [39, 40]. As a result, only some biological characteristics of rice hull development have been investigated. The recent development of iTRAQ has allowed full-scale alterations of protein expression profiles to be examined under particular defined conditions [24–26, 41, 42]. To reduce the impact of individuals, we pooled rice hull samples from various development stages in the iTRAQ study. Our results showed that the label-based iTRAQ quantitative proteomic approach successfully detected and quantified the overall protein profile alternations in the booting, flowering, and milk-ripe stages of rice hull development. The unsupervised hierarchical clustering analysis indicated that the protein profiles of the flowering and milk-ripe stages were more similar to each other than they were to the protein profile of the booting stage (Fig 2A). When GO terms were assigned to the differentially expressed proteins in the three development stages, we found that the GO term numbers in the booting and flowering stages were more similar than either of them were to GO term numbers in the milk-ripe stage. This finding suggested that the level of activity in the rice hull proteome was similar in the booting and flowering stages compared with in the milk-ripe stage. The overall gene ontology analysis showed that GO terms of biological process and cellular component were much more diverse than molecular function terms in three growth stages of rice hull. It indicated that molecular function terms would be more likely to distinguish the characterization of rice hull at various growth stages.

Molecular function was highlighted to help understand the protein activities at the molecular level. Nine GO terms under the molecular function category were enriched and differentially overexpressed at various growth stages. We identified 25 candidate proteins annotated with five GO terms; two for oxidoreductase activity (GO:0016620 and GO:0016903), the others for transaminase activity (GO:0008483), transferase activity (GO:0016769), and flavin adenine dinucleotide binding (GO:0050660). The unannotated protein (Q7X8A1) was overexpressed especially in the flowering stage, indicating that this protein may be an important regulatory protein that distinguishes the flowering stage from the other growth stages. A putative uncharacterized protein (B8BF94) and ALDH (Q9FPK6) were overexpressed only in the milk-ripe stage, suggesting that these proteins may be crucial in the milk-ripe stage of rice hull development. Some proteins, for example, GAPC3 (Q6K5G8) and Aldh2b (Q9FRX7), were overexpressed in the flowering and milk-ripe stages, indicating that they may play pivotal roles in these two growth stages. The 25 identified candidate proteins may be major contributors to the heterogeneity of the rice hull proteome at various growth stages.

Pathways regulated by differentially expressed proteins offered an opportunity to observe the dynamic cell behavior in the rice hull development. In each cell, signal transduction and regulatory systems transmit information from the cell to its environmental status, thereby controlling the expression levels of every protein in the cell. UniPathway [38], a manually curated pathway database, was used to analyze the biology mechanisms of the differentially expressed proteins in the proteomic analysis to determine the dynamic cell behavior during rice hull development. GAPC3 (Q6K5G8) was upregulated in the glycolysis pathway in the flowering and milk-ripe stages compared with in the booting stage. Glycolysis is a pivotal process in the breakdown of glucose to generate energy and to provide biosynthetic precursors for cellular materials [43]. Glycolysis also plays an important role in maintaining cell division and controlling cell proliferation [44, 45]. The high expression of GAPC3 in the flowering and milk-ripe stages suggested that this protein may play a pivotal role in the glycolysis pathway during rice hull development. Sucrose metabolism is known to play crucial roles in development, stress response, and yield formation [46]. Deficient sucrose has been reported to cause an overall decrease in cell wall polymers [47]. A probable sucrose-phosphate synthase (Q0JGK4) was upregulated in the flowering and milk-ripe stages, suggesting that it might be an important regulator in sucrose biosynthesis. Other pathways such as light-dependent chlorophyll biosynthesis and oxylipin biosynthesis were also found to be related to rice hull development. Chlorophyll biosynthesis plays essential roles in photosynthesis [48], and oxylipin biosynthesis plays important roles in adaptation to photo-oxidative stress [49]. The rice grain thickens and hardens during the milk-ripe stage and, at the same time, the outer covering changes from green to yellow. Upregulation of the magnesium-protoporphyrin IX monomethyl ester [oxidative] cyclase (Q9SDJ2) in the flowering stage suggested that it may be involved in chlorophyll accumulation and chloroplast development. Three chlorophyll biosynthesis pathways related proteins involved to magnesium-protoporphyrin IX monomethyl ester [oxidative] cyclase (Q9SDJ2), ChlD (Q6ATS0) and Ch1I (Q53RM0), were significantly downregulated in the milk-ripe stage. These proteins, especially magnesium-protoporphyrin IX monomethyl ester [oxidative] cyclase (Q9SDJ2) identified by qRT-PCR, may directly influence the biosynthesis of chlorophyll and eventually change the chlorophyll content at the different developmental stages. To our knowledge, this is the first study to use the well established quantitative iTRAQ label-based technology for proteomic analysis of rice hull during its development. We identified 25 differentially expressed proteins that may be associated with various growth stages. GO terms and pathways with related protein expression alterations may provide new insights into the mechanisms of rice hull development. The results have provided new information for understanding the molecular characterization of rice hull development at various growth stages.

## Conclusions

This study provides a global profiling of changes in the rice hull proteome during development. GO and pathways analysis of the differentially expressed proteins revealed dynamic cell behavior during hull development, and provided new insights into the molecular mechanisms of rice hull development at various growth stages.

## Supporting Information

S1 Fig Cellular function analysis of differentially expressed proteins in three growth stages of rice hull. P1, booting stage; P2, flowering stage; P3, milk-ripe stage
(GIF)

S2 Fig Biological process analysis of differentially expressed proteins in three growth stages of rice hull. P1, booting stage; P2, flowering stage; P3, milk-ripe stage.
(GIF)

S1 Table Quantitative changes of differentially expressed proteins in rice hulls at various growth stages. (XLS)

S2 Table GO classification of differentially expressed proteins involved in booting stage (PK1), flowering stage (PK2) and milk-ripe stage (PK3). (XLS)
