# Supplementary figures and images for: Differential Proteomic Analysis Using iTRAQ Reveals Alterations in Hull Development in Rice (Oryza sativa L.)
Source: PLoS One. 2015 Jul 31;10(7):e0133696. doi: 10.1371/journal.pone.0133696 (PMC4521873; doi:10.1371/journal.pone.0133696)

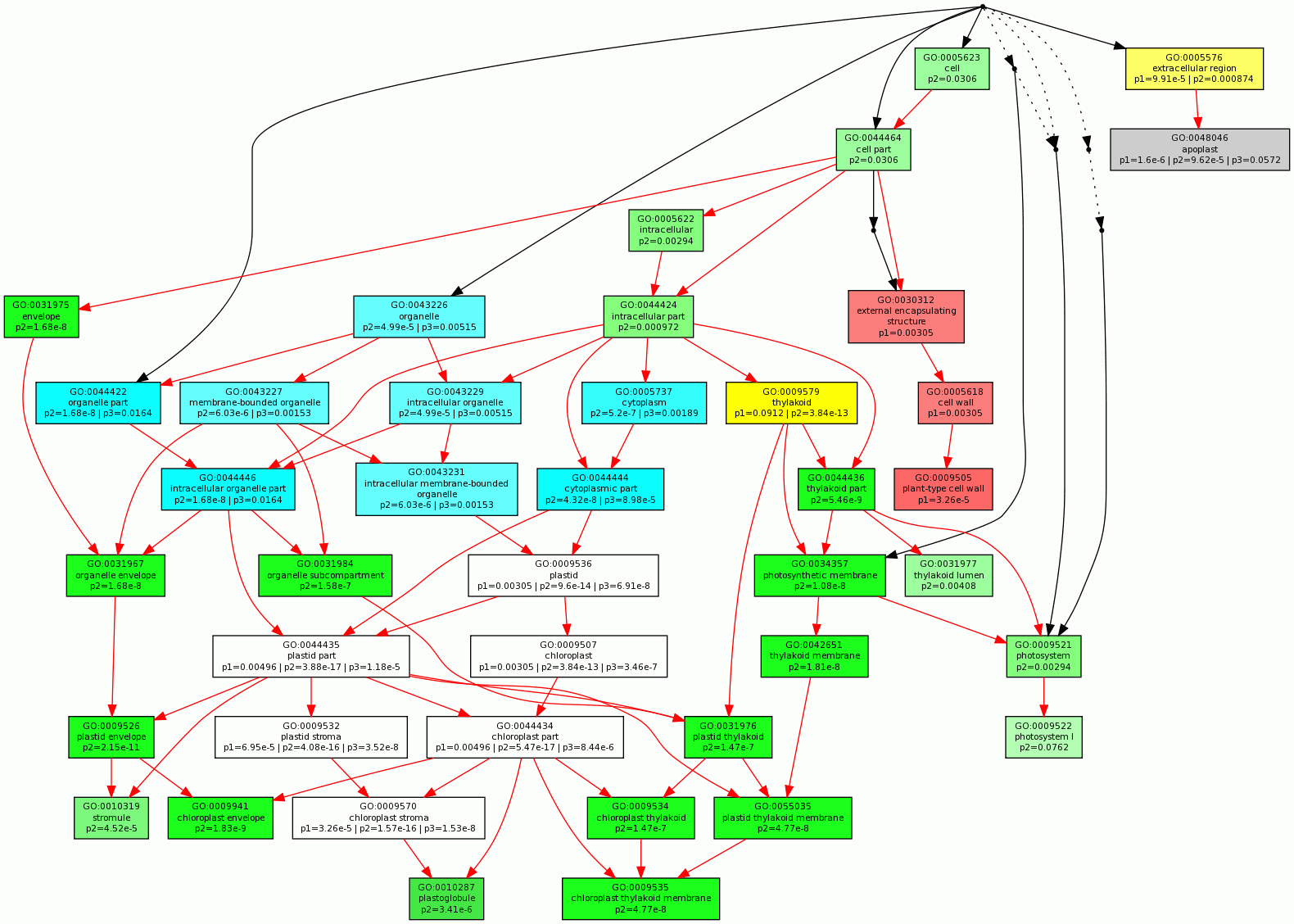

Supplement: S1 Fig — P1, booting stage; P2, flowering stage; P3, milk-ripe stage (GIF) [file pone.0133696.s001.gif]

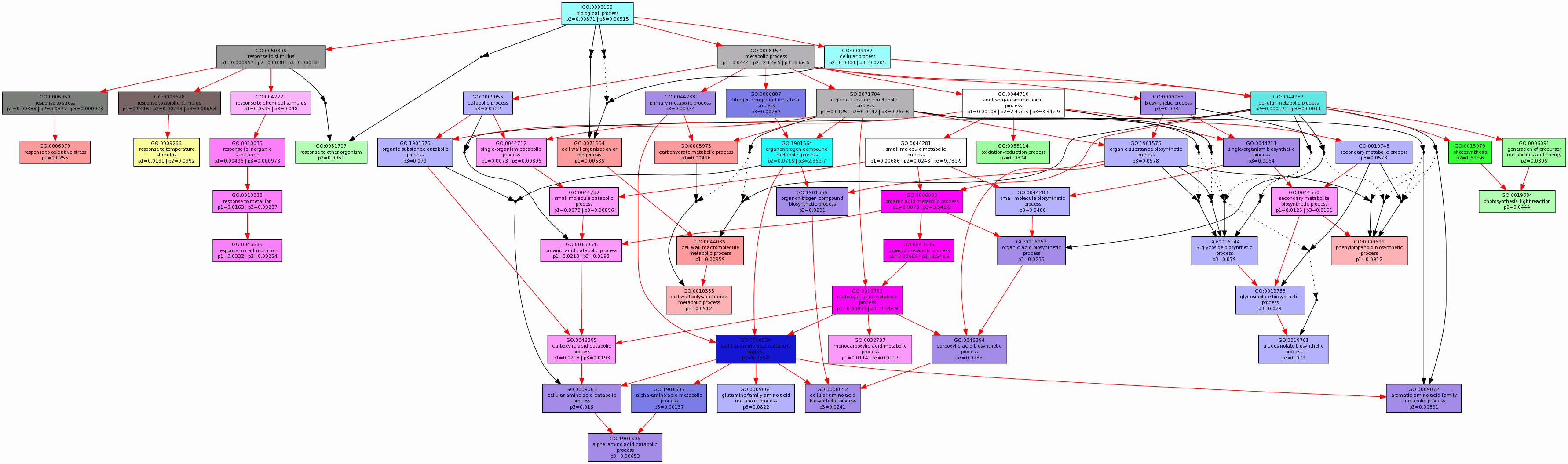

Supplement: S2 Fig — P1, booting stage; P2, flowering stage; P3, milk-ripe stage. (GIF) [file pone.0133696.s002.gif]
